# Impact of demography on linked selection in two outcrossing Brassicaceae species

**DOI:** 10.1002/ece3.5463

**Published:** 2019-08-13

**Authors:** Tiina M. Mattila, Benjamin Laenen, Robert Horvath, Tuomas Hämälä, Outi Savolainen, Tanja Slotte

**Affiliations:** ^1^ Department of Ecology and Genetics University of Oulu Oulu Finland; ^2^ Science for Life Laboratory, Department of Ecology, Environment, and Plant Sciences Stockholm University Stockholm Sweden; ^3^ Biocenter Oulu University of Oulu Oulu Finland; ^4^ Present address: Department of Organismal Biology Uppsala University Uppsala Sweden; ^5^ Present address: Department of Plant and Microbial Biology University of Minnesota Twin Cities St. Paul MN USA

**Keywords:** demography, distribution of fitness effects, linked selection, neutral genetic diversity, purifying selection, recombination

## Abstract

Genetic diversity is shaped by mutation, genetic drift, gene flow, recombination, and selection. The dynamics and interactions of these forces shape genetic diversity across different parts of the genome, between populations and species. Here, we have studied the effects of linked selection on nucleotide diversity in outcrossing populations of two Brassicaceae species, *Arabidopsis lyrata* and *Capsella grandiflora*, with contrasting demographic history. In agreement with previous estimates, we found evidence for a modest population size expansion thousands of generations ago, as well as efficient purifying selection in *C. grandiflora*. In contrast, the *A. lyrata* population exhibited evidence for very recent strong population size decline and weaker efficacy of purifying selection. Using multiple regression analyses with recombination rate and other genomic covariates as explanatory variables, we can explain 47% of the variance in neutral diversity in the *C. grandiflora* population, while in the *A. lyrata* population, only 11% of the variance was explained by the model. Recombination rate had a significant positive effect on neutral diversity in both species, suggesting that selection at linked sites has an effect on patterns of neutral variation. In line with this finding, we also found reduced neutral diversity in the vicinity of genes in the *C. grandiflora* population. However, in *A. lyrata* no such reduction in diversity was evident, a finding that is consistent with expectations of the impact of a recent bottleneck on patterns of neutral diversity near genes. This study thus empirically demonstrates how differences in demographic history modulate the impact of selection at linked sites in natural populations.

## 1 INTRODUCTION

The relative roles of selection and genetic drift in shaping genome‐wide variation are of great interest in evolutionary genetics. The genetic variation carried by a species is produced by mutation, but in finite populations some neutral variants are lost by chance while others may increase in frequency through genetic drift. The strength of genetic drift is determined by the effective size of the population, *N*_e_ (Fisher, 1930; Wright, 1931). In contrast, negative selection decreases and positive selection increases the frequency of the selected variant deterministically reducing variation at the site under selection. Due to linkage disequilibrium, the associated reduction in variation is not limited to the site under selection itself but extends to nearby sites. The effect of selection at linked sites (“linked selection”) can be a result of either selection against deleterious mutations, known as background selection (Charlesworth, Morgan, & Charlesworth, 1993), or selection favoring advantageous mutations, that is genetic hitchhiking (Maynard Smith & Haigh, 1974). Since a large proportion of new mutations occurring in functional regions have fitness effects (Eyre‐Walker & Keightley, 2007), linked selection is likely of wide importance for determining levels of polymorphism within populations (Charlesworth, 1994; Charlesworth, Charlesworth, & Morgan, 1995; Kim & Stephan, 2002). Linked selection has also been suggested to account for the narrow range of diversity across species despite potentially large differences in census population sizes (Corbett‐Detig, Hartl, & Sackton, 2015; Gillespie, 2001). However, the impact of linked selection does not seem to be sufficient to fully explain variation in diversity levels among species (Coop, 2016). Nevertheless, understanding the impact of linked selection, especially in the form of background selection, is important for inference of adaptation, since genomic regions experiencing different magnitudes of background selection are expected to harbor different baseline levels of diversity (Burri et al., 2015; Comeron, 2014, 2017; Elyashiv et al., 2016; Huber, DeGiorgio, Hellmann, & Nielsen, 2016). Furthermore, background selection causes distortion of gene genealogies such that site frequency spectra may be expected to exhibit an excess of rare alleles (Nordborg, Charlesworth, & Charlesworth, 1996; Zeng & Charlesworth, 2011). If not properly accounted for, linked selection may thus give rise to a false signal of population size expansion in demographic inference (Ewing & Jensen, 2016) and result in incorrect model selection (Schrider, Shanku, & Kern, 2016). As linked selection can affect a large proportion of the genome (Pouyet, Aeschbacher, Thiéry, & Excoffier, 2018; Woerner, Veeramah, Watkins, & Hammer, 2018), characterizing its effects is of great importance.

Since the extent over which selection at linked sites can affect polymorphism depends on the amount of linkage disequilibrium, linked selection causes a positive correlation between diversity and recombination rate (Hudson & Kaplan, 1995; Nordborg et al., 1996). In regions with high recombination rates, the effect of selection at linked sites is less pronounced (Charlesworth et al., 1993; Kaplan, Hudson, & Langley, 1989; Thomson, 1977), a pattern observed in multiple species (reviewed by Cutter & Payseur, 2013; Slotte, 2014). The first empirical evidence supporting an important effect of linked selection on patterns of genetic diversity was in *Drosophila melanogaster*, where Begun and Aquadro (1992) found a correlation between diversity and recombination rate. However, recombination may have a mutagenic effect itself that can lead to such a correlation even in the absence of linked selection. This effect can be tested by taking into account mutation rate variation along the genome, for example by using synonymous divergence as a proxy (Begun & Aquadro, 1992; Hellmann, Ebersberger, Ptak, Pääbo, & Przeworski, 2003). Other factors may also impact the diversity distribution along the genome and the effect of linked selection. For example, the impact of linked selection may depend on variation in the density of functional sites, which may further be correlated with recombination. If recombination rate and gene density are correlated (Flowers et al., 2011; Slotte, 2014), the relationship between recombination rate and diversity may be more complex.

Furthermore, a difference in the pattern and magnitude of linked selection is expected in populations with different demographic histories, as for instance in humans and chimpanzees (Pfeifer & Jensen, 2016). At equilibrium, the rate of random loss of variation due to genetic drift is lower in populations with a large *N*_e_, and hence, they can carry more variation (Wright, 1931). In addition, selection efficacy depends on the effective population size and selection is only efficient relative to drift if |*N*_e_ *s|* > 1, where *s* is the selection coefficient. Thus, variants with a small fitness effect behave neutrally in small populations (Ohta, 1973). Similarly, at equilibrium, the strength of background selection scales with *N*_e_, with stronger linked selection in large populations (Nordborg et al., 1996). However, the magnitude of linked selection may vary depending on the combination of population parameters such as selection coefficient and *N*_e_ (Nam et al., 2017). Furthermore, if population size fluctuates over time, alleles of different age may experience different magnitude of linked selection, as was found in a study that contrasted diversity in maize and teosinte (Beissinger et al., 2016). Thus, under nonequilibrium demographic models, diversity patterns may differ greatly from predictions based on classical models assuming equilibrium (Torres, Stetter, Hernandez, & Ross‐Ibarra, 2019). An improved understanding of the interplay between demographic history and linked selection is thus of broad importance.

To empirically investigate genome‐wide patterns of linked selection in populations with contrasting nonequilibrium demographic histories, we studied genome‐wide diversity patterns in two herbaceous outcrossing Brassicaceae species, *Arabidopsis lyrata* and *Capsella grandiflora*. We chose these species because they have similar mating systems (both are self‐incompatible outcrossers) and genome structure, and because previous research suggests considerable differences in the demographic histories of some populations. Indeed, the recent colonization history of *A. lyrata* especially in northern Europe has likely been associated with strong population bottlenecks or effective population size reduction (Mattila, Tyrmi, Pyhäjärvi, & Savolainen, 2017; Pyhäjärvi, Aalto, & Savolainen, 2012; Ross‐Ibarra et al., 2008; Savolainen & Kuittinen, 2011), which is in contrast to the large and relatively stable population of *C. grandiflora* (Douglas et al., 2015; Slotte, Foxe, Hazzouri, & Wright, 2010; Slotte et al., 2013; St. Onge, Källman, Slotte, Lascoux, & Palmé, 2011). Hence, this species pair offers an opportunity to investigate the interplay between demographic history and linked selection. Here, we first revisit the demographic analysis, investigate patterns of genome‐wide variation, and quantify the magnitude of linked selection using whole‐genome resequencing data from natural populations. Our primary assumption was that linked selection is stronger in the population with a larger long‐term effective population size. We also discuss our results in the light of expected effects of linked selection after population size change.

## 2 MATERIALS AND METHODS

### 2.1 Sequence datasets

Raw Illumina sequence data for 12 *A. lyrata* individuals originating from Spiterstulen, Norway (61°38′N, 8°24′E), were included in the analyses (data from Mattila et al., 2017; Hämälä, Mattila, & Savolainen, 2018). For *C. grandiflora*, Illumina whole‐genome resequencing data for 12 individuals, originally collected from a population near the village of Koukouli, Zagory, Greece (39°52′N, 20°46′E), were obtained from Steige, Laenen, Reimegård, Scofield, and Slotte (2017). The analyzed *C. grandiflora* dataset was randomly subsampled from the original set of 21 individuals, to ensure balanced datasets from both species. Detailed sampling and sequencing information is available in the original publications.

### 2.2 Sequence processing and reference mapping

Raw sequence data were preprocessed and mapped to *A. lyrata* Ensembl plant version 1.0.29 (Hu et al., 2011; Kersey et al., 2014) and *Capsella rubella* v. 1.0 (Slotte et al., 2013) reference genomes. We chose to use the *C. rubella* genome as it is considerably less fragmented than currently available *C. grandiflora* assemblies, and because *C. rubella* and *C. grandiflora* are very closely related (split time estimated to <200 kya; Koenig et al., 2019; Slotte et al., 2013; approximately 84% of polymorphisms in *C. rubella* are shared with *C. grandiflora*; Foxe et al., 2009). Our sequence processing and mapping approach followed a pipeline developed in Mattila et al. (2017), with slight modifications. Briefly, sequence data were first processed with Trimmomatic 0.32 (Bolger, Lohse, & Usadel, 2014) in paired‐end mode with the TrueSeq2 paired‐end adapters and the following options—ILLUMINACLIP/TruSeq2‐PE.fa:5:20:10 ‐LEADING 10 ‐TRAILING 10 ‐SLIDINGWINDOW 10:20 ‐MINLEN 50. Both read pairs were required to pass the trimming for further analysis.

Reads were then mapped to reference genomes using bwa‐mem (Li, 2013; Li & Durbin, 2009) with options ‐M ‐r 1. Duplicates were marked, read groups added, and alignment statistics calculated with PICARD TOOLS v. 1.113 (http://broadinstitute.github.io/picard). Overlapping read pairs were trimmed using BamUtil v. 1.0.13 (https://genome.sph.umich.edu/wiki/BamUtil) using the option unmapped. Indels were marked and realigned with Genome Analysis Toolkit v. 3.2.2 (De Pristo et al., 2011). We utilized the pipeline tool STAPLER (Tyrmi, 2018) for automatic creation and management of scripts for running processes on a computer cluster. The proportion of mapped reads was very similar for both *C. grandiflora* and *A. lyrata* (approximately 90%). Mapping statistics and additional summary per individual data are available at supporting information S2.

### 2.3 Quantifying the patterns of polymorphism

Site frequency spectra (SFS) for both species were calculated using ANGSD v. 0.913‐56‐g5b7889d (Korneliussen, Albrechtsen, & Nielsen, 2014; Nielsen, Korneliussen, Albrechtsen, Li, & Wang, 2012). The estimated SFS were used as priors for estimating nucleotide diversity (*π*) (Nei & Li, 1979) and Watterson's theta (Watterson, 1975) as implemented in ANGSD. Per base pair estimates for the diversity estimates per window were obtained by dividing the estimate by the number of sites analyzed per window. For all analyses, we used the genotype likelihood calculation method from McKenna et al. (2010). Estimates were calculated separately for 0‐fold, 4‐fold, and intergenic sites. The 0‐fold and 4‐fold degenerate sites were extracted from the reference genome and the gene annotation using the script NewAnnotateRef.py (Williamson et al., 2014) for both species. For *A. lyrata*, exon annotation rows were removed from the original gff3 file before running the annotation script and all sites with no annotation in the Ensembl version 1.0.29 were assigned as intergenic. A bed file with the noncoding (intergenic) regions was subtracted from the.gff3 annotation files with BEDTools v2.26.0 (Quinlan & Hall, 2010) subtract tool.

For diversity calculations all repeat annotated regions, sites with fixed heterozygosity (allowing 20% of missing data) ±200 base pairs and conserved noncoding regions (Haudry et al., 2013) were masked in both species. Additionally, regions aligning against *Arabidopsis thaliana* organelle genomes or regions with discordant sequence between the NCBI and Ensembl reference genomes were excluded from *A. lyrata* data. Species alignments were obtained from Ensembl Compara for *A. lyrata*.

Putative paralogous regions were excluded based on raw SNP calls showing a pattern of fixed heterozygotes at the population sample following the procedure previously described in Mattila et al. (2017). We called biallelic SNPs (excluding indels and complex events) using freebayes v1.0.2‐33‐gdbb6160 (Garrison & Marth, 2012) with minimum coverage 5, mapping quality 30, base quality 20 (‐‐min‐coverage 5 ‐q 20 ‐m 30 ‐i ‐u ‐v ‐X).

The following filtering settings were used for the diversity calculation in ANGSD: ‐remove_bads ‐unique_only ‐minMapQ 30 ‐minQ 20 ‐only_proper_pairs 1 ‐trim 0. For both species, mean depth plus 3 * *SD* was used as individual maximum depth. We further required 80% of data presence ensuring that the analyses are not based on single or a few individuals. This filters out poorly covered regions some of which may contain structural or indel variants. Such regions may suffer from allele dropout biasing diversity estimates. Doing so we potentially exclude some fast‐evolving regions but we were not, however, able to cover the variation in these regions with the current dataset. Moreover, this filter should not bias comparisons between species.

### 2.4 Demographic inference

While previous studies have investigated demographic history in both *A. lyrata* and *C. grandiflora*, earlier studies have not always used sets of sites that are robust to the impact of linked selection. We therefore opted to revisit this question and conduct demographic inference using our data. To do so, we used the diffusion approximation method implemented in the Python library ∂*a*∂*i* (Gutenkunst, Hernandez, Williamson, & Bustamante, 2009). Five different demographic models covering a variety of possible single population demographic histories were considered: constant, two‐epoch, three‐epoch, growth, and bottlegrowth (Figure S1). For each model, ∂*a*∂*i* was run with 500 starting points followed by a grid search around the best likelihood values. The best model was chosen using the Akaike information criterion (AIC). For demographic inference, we used site frequency spectra obtained for intergenic sites at least 2,000 bp from genes (using BEDtools slop) and within the upper quartile of recombination rates, as measured in 50 kb windows. Such genomic regions have previously been suggested to be least affected by linked selection and thus useful for demographic inference (Coop, 2016). These sites are denoted by intergenic_high‐rec_ hereafter. After filtering, all eight chromosomes were represented in the *C. grandiflora* dataset, but in the *A. lyrata* dataset windows only from chromosomes 1, 2, 3, 5, and 7 were included. Unfolded SFS and 100 bootstrap SFS were estimated with ANGSD as described above. We opted for using the folded SFS for demographic inference to avoid potential biases due to ancestral state misinference. Confidence intervals for demographic parameter estimates were calculated from analyses of 100 bootstrap replicates.

### 2.5 Divergence

Divergence at 4‐fold and 0‐fold sites (*d*_4_ and *d*_0_) was estimated from a whole‐genome alignment of *C. rubella—A. lyrata* (Steige et al., 2017). Window‐based divergence (50 kb) was calculated by dividing the sum of divergent sites by the total number of sites at which divergence could be reliably assessed within each window, using the BEDtools map option.

### 2.6 Recombination rate estimates

The recombination map for *A. lyrata* was obtained from Hämälä, Mattila, Leinonen, Kuittinen, and Savolainen (2017). For *C. grandiflora*, we used the genetic map from Slotte, Hazzouri, Stern, Andolfatto, and Wright (2012) which is based on an interspecific F2 mapping population between *C. grandiflora* and *C. rubella*. As the F2 population did not exhibit signs of genetic incompatibilities, and as the two species are very closely related (split time < 200 kya; Koenig et al., 2019; Slotte et al., 2013), we expect this genetic map to provide a reasonable estimate of broad recombination rate variation in *C. grandiflora*. Maps were thinned to include only markers at least 10 kb apart by dropping all markers for which distance to the previous included marker was less than the given threshold, starting from the beginning of the chromosome. In addition, ascending order of marker physical positions in relation to genetic positions was required. Hence, a large inverted region in *A. lyrata* chromosome 7 was excluded (approximately 4.3 Mb sized region). Recombination rates per base pair (Figures S2 and S3) were estimated from the recombination map using R (R Core Team, 2016) function smooth spline interpolation with smoothing parameter 0.7.

### 2.7 Codon usage bias

We quantified codon usage bias as the effective number of codons, estimated using a method accounting for background base composition (Novembre, 2002). Base composition corrected effective number of codons (*N*_c_') was calculated as implemented in the program ENCprime (https://github.com/jnovembre/ENCprime).

### 2.8 Transposable element content

Transposable element content was estimated using RepeatMasker (Chen, 2004) using an *Arabidopsis* TE database for both species. From the raw repeat annotation, simple repeats were excluded to limit the analysis to transposable element repeats only.

### 2.9 Distribution of fitness effects and estimate of positive selection

We estimated the distribution of fitness effects of new mutations and the proportion of substitutions fixed by positive selection at 0‐fold degenerate sites using DFE‐alpha v.2.15 (Eyre‐Walker & Keightley, 2009; Keightley & Eyre‐Walker, 2007). Mutations at these sites are amino acid changing and may thus have an effect on fitness. For these analyses, we considered 4‐fold degenerate sites as neutral. Since demographic changes may have an effect on DFE estimation, we estimated DFE taking into account the demographic history estimated from the neutral site category as implemented in DFE‐alpha (Keightley & Eyre‐Walker, 2007). The population‐based selection coefficients (*N*_e_ *s*) were categorized in three groups: nearly neutral (*N*_e_ *s* < 1), intermediate (1 < *N*_e_ *s* < 10), and strongly deleterious (*N*_e_ *s* > 10). Divergence at 4‐fold and 0‐fold sites was calculated as described above and used to estimate the proportion of positively selected 0‐fold degenerate fixations (*α*), and *ω*_a_, a measure of the proportion of positively selected 0‐fold fixations relative to neutral divergence (Gossmann et al., 2010). SFS and 100 bootstrap replicates for both site categories were estimated with ANGSD as described above. The run with the highest likelihood out of five independent runs was retained. This procedure was repeated for the 100 bootstrap SFS to get 95% confidence intervals for the estimated parameters.

### 2.10 Factors explaining neutral diversity

In order to assess the impact of linked selection on the level of neutral diversity, we fit a model with diversity at 4‐fold degenerate sites (*π*_4_) as response variable and recombination rate and the number of functional sites, measured as the number of base pair annotated as part of an exon (exonic bp), as explanatory variables using the “glm” function in R. To account for mutation rate and other factors possibly correlated with diversity, we included divergence at 4‐fold degenerate sites (*d*_4_), codon usage bias (*N*_c_'), and transposable element (TE)% in the models as additional explanatory variables. All variables were calculated in 50 kb nonoverlapping windows. The additional covariates were included in the model since recombination rate has also been shown to correlate with transposable element (TE) density (Bartolomé, Maside, & Charlesworth, 2002; Rizzon, Marais, Gouy, & Biémont, 2002; Tenaillon, Hollister, & Gaut, 2010) and may also affect the base composition through selection for optimal codon usage or biased gene conversion (Galtier, Piganeau, Mouchiroud, & Duret, 2001; Plotkin & Kudla, 2011) with a possible effect on neutral diversity. Hence, inclusion of these other confounding factors is pivotal when dissecting the forces shaping diversity.

To allow reasonably robust divergence estimates, windows with <500 sites to estimate *π*_4_ (callable sites) and *d*_4_ (aligned sites) were excluded. In addition, we excluded windows with *d*_4_ > 0.3 and recombination rate > 9 × 10^–6^ cM/bp (single outlier peak in *Capsella*) which might indicate alignment or genotyping errors, respectively. A model selection procedure was applied to evaluate the importance of each explanatory variable in the model using stepAIC from R package MASS (Venables & Ripley, 2002) with Bayesian information criterion (BIC). All variables were centered and scaled prior to analysis. The effect of multicollinearity of the explanatory variables was evaluated with variance inflation factor (vif) analysis using the vif function of the R car package (Fox & Weisberg, 2011) with √vif < 2 was required for each variable. Divergence at 0‐fold degenerate sites (*d*_0_) was excluded from the model due to high variance inflation in *A. lyrata*. A single outlier in the *A. lyrata* dataset with exceptionally high diversity (which may indicate paralogous mapping) was removed.

### 2.11 Diversity around genes

Diversity was first calculated in 1 kb windows for intergenic sites. The distance to the nearest gene of each window was calculated with BEDTools closest option. The recombination maps were used to convert the physical distances into genetic distance (cM). The genome‐wide ratio of per window and global intergenic diversity (*π*/mean *π*) was estimated with the smooth.spline R function from the observed window‐based data, and 95% confidence regions were generated using 100 bootstrap datasets. These distributions were used to calculate significance in each distance category.

## 3 RESULTS

### 3.1 *A. lyrata* and *C. grandiflora* differ in levels and distribution of diversity

After quality filtering and region masking, we retained 60.5 million sites in *A. lyrata* and 61.8 million sites in *C. grandiflora* for analyses of polymorphism levels. Of these sites, 1.3 million were variable in the *A. lyrata* population and 4.3 million in the *C. grandiflora* population. Without quality filtering, there were (the masking settings were not changed) 73.4 million sites in *A. lyrata*, 2.1 million of which were variable, and 70 million sites in *C. grandiflora*, 5.2 million of which were variable. The largest difference after filtering was observed at intergenic sites (Table 1).

**Table 1:** The number of sites analyzed with (F) and without filtering (NF) for 0‐fold, 4‐fold, intergenic (i), and intergenic_high‐rec_ sites

Species	Site category	Sites_F_	Variable sites_F_	% variable_F_	Sites_NF_	Variable sites_NF_	% variable_NF_
*A. lyrata*	0‐fold	18,230,869	184,038	0.01	19,644,419	232,196	0.01
	4‐fold	4,177,306	135,645	0.03	4,493,448	155,655	0.03
	intergenic_high‐rec_	2,466,049	60,905	0.02	3,195,448	107,053	0.03
	intergenic	38,110,094	1,035,010	0.03	49,292,193	1,708,184	0.03
*C. grandiflora*	0‐fold	19,755,662	467,158	0.02	20,348,623	511,293	0.03
	4‐fold	4,599,325	403,789	0.09	4,730,965	424,878	0.09
	intergenic_high‐rec_	2,428,095	255,999	0.11	3,271,623	362,859	0.11
	intergenic	37,541,515	3,391,602	0.09	44,949,263	4,298,691	0.10

The average nucleotide diversity at 4‐fold degenerate sites (*π*_4_) was higher for the *C. grandiflora* population than for the *A. lyrata* population (0.0224 vs. 0.0101, respectively; Table 2, Wilcoxon rank sum test, *W* = 839,980, *p*‐value < 0.001). Higher *π* in *C. grandiflora* than in *A. lyrata* was also observed at the other site categories. In *A. lyrata*, the highest nucleotide diversity (*π*) was observed at 4‐fold degenerate sites while in *C. grandiflora* intergenic sites with additional filtering (intergenic_high‐rec_ sites that were distant from genes, in regions with high recombination rates) had the highest *π* (Table 2). Watterson's *θ* estimates were slightly higher than *π* estimates in the *C. grandiflora* dataset while in *A. lyrata* the opposite pattern was observed. The ratio of 0‐fold to 4‐fold degenerate nucleotide diversity (*π*_0_/*π*_4_) was 0.2187 for the *C. grandiflora* population while in the *A. lyrata* population it was 0.2846 (Table 2), which may indicate stronger purifying selection in the *C. grandiflora* population or faster recovery of *π*_0_ after a population bottleneck in the *A. lyrata* population (Comeron, 2017).

**Table 2:** Diversity estimates for 0‐fold, 4‐fold, intergenic (i), and intergenic_high‐rec_ (i2) sites (95% C.I. based on 10,000 bootstrapped datasets in parentheses) in *Arabidopsis lyrata* and *Capsella grandiflora* populations

Species	*θ*_W0_	*π*_0_	*θ*_W4_	*π*_4_	*θ*_Wi_	*π*_i_	*θ*_Wi2_	*π*_i2_	*π*_0_/*π*_4_
*A. lyrata*	0.0024 (0.0024–0.0025)	0.0029 (0.0028–0.003)	0.0081 (0.0078–0.0083)	0.0101 (0.0098–0.0104)	0.0071 (0.0069–0.0072)	0.0084 (0.0082–0.0086)	0.006 (0.0057–0.0064)	0.0073 (0.0068–0.0076)	0.2846 (0.279–0.2903)
*C. grandiflora*	0.0058 (0.0057–0.0059)	0.0048 (0.0046–0.0049)	0.0234 (0.0228–0.024)	0.0224 (0.0217–0.023)	0.0234 (0.023–0.0238)	0.0204 (0.0201–0.0207)	0.0276 (0.0269–0.0281)	0.0234 (0.0228–0.0243)	0.2187 (0.2156–0.2218)

Note

*θ*_W_ = Watterson's theta, *π* = nucleotide diversity

**Figure 1:**
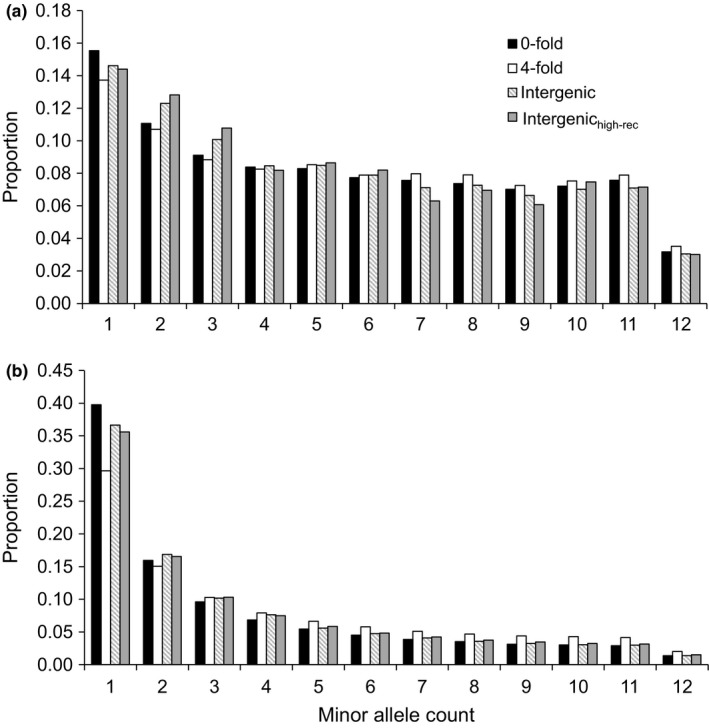
Folded site frequency spectra of 0‐fold (black), 4‐fold (white), intergenic (striped), and intergenic_high‐rec_ sites for (a) *A. lyrata* and (b) *C. grandiflora*. Frequency categories standardized by the total number of polymorphic sites within each site category. Note the different *y*‐axes scales

The two datasets also differed with respect to their site frequency spectra. Specifically, our *A. lyrata* population had a higher proportion of SNPs at intermediate frequency in comparison with *C. grandiflora* (Figure 1), likely reflecting differences in the demographic history of the study populations. In addition to differences due to demographic history, a higher proportion of low frequency variants at sites affected by background selection is expected (Zeng & Charlesworth, 2011). Hence, the highest proportion of low frequency variants were expected at 0‐fold sites and lowest at intergenic sites. However, both species had the highest proportion of singletons at 0‐fold degenerate sites and the lowest at 4‐fold degenerate sites, while the frequency of singletons was intermediate at intergenic sites. Moreover, the frequency of doubletons was highest at the intergenic sites (Figure 1).

### 3.2 Demographic histories

**Figure 2:**
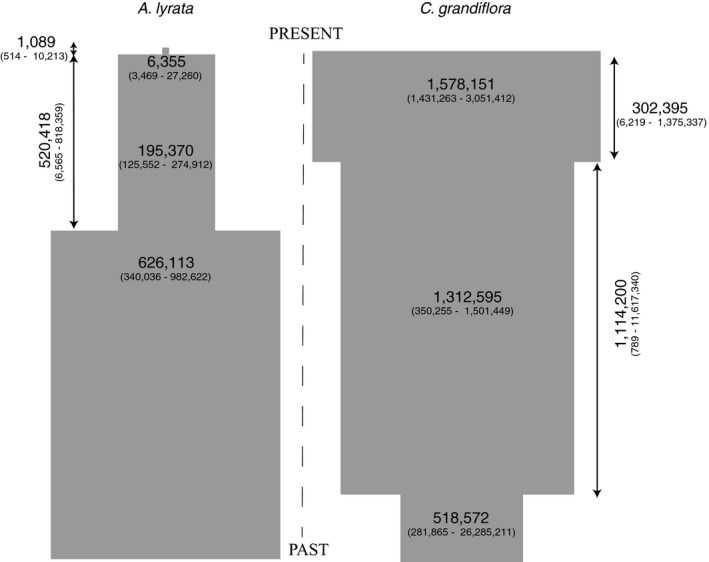
Demographic parameter estimates (95% C.I. in parentheses) for *A. lyrata* and *C. grandiflora*. Time estimates are in generations and effective population sizes in diploid individuals

We next assessed the demographic history of these two populations using SFS estimated from the intergenic_high‐rec_ sites. The demographic inference suggests that the best model for our *A. lyrata* population was a three‐epoch model with strong effective population size decrease from 626,000 (340,000–983,000, 95% C.I.) to 195,000 (126,000–275,000, 95% C.I.). We estimated that the bottleneck length was approximately 520,000 (7,000–818,000, 95% C.I.; Figure 2; Table S1) generations. The population size shrunk further to 6,000 (3,000–27,000, 95% C.I.) approximately 1,000 (500–10,000, 95% C.I.) generations ago (Figure 2; Table S1). Our model selection procedure was not able to separate between the population expansion model and the three‐epoch model for our *C. grandiflora* dataset (AIC difference < 2, Table S1, Burnham & Anderson, 2004) but they both suggest a modest population size increase in the past. With the three‐epoch model, we estimated a stepwise effective population size increase from an ancestral population size of 519,000 (282,000–26,285,000, 95% C.I.) to 1,313,000 (350,000–1,501,000, 95% C.I.) that occurred approximately 1.4 million generations ago and persisted over approximately 1,114,000 generations (800–11,617,000, 95% C.I.; Figure 2; Table S1). A second slight increase to 1,578,000 (1,431,000–3,051,000, 95% C.I.) was estimated to have occurred approximately 302,000 (6,000–1,375,000, 95% C.I.) generations ago (Figure 2; Table S1). Our timing estimates and the ancestral population size estimate especially for *C. grandiflora* contain considerable uncertainty (Figure 2; Table S1), but nevertheless suggest that there have been more recent drastic population size changes in the *A. lyrata* population than in the *C. grandiflora* population. Hence, a difference in the state of recovery after population size change is expected.

### 3.3 Weaker purifying selection in *A. lyrata*

The difference in the ratio of *π*_0_ and *π*_4_ may reflect a difference in the genome‐wide efficacy of purifying selection between our *C. grandiflora* and *A. lyrata* populations. To assess purifying selection in nonequilibrium populations, we estimated the distribution of fitness effects (DFE) of new mutations at 0‐fold degenerate sites, while accounting for nonequilibrium demographic history using a simple demographic model as implemented in DFE‐alpha. In general, the estimated demography was in line with the previous results; for the *C. grandiflora* population, we estimated a slight effective population size increase and for the *A. lyrata* population a strong reduction in the effective population size (Table 3). The estimated DFEs were strongly leptokurtic for both species, especially for *A. lyrata* (Table 3). Such gamma distributions with low beta estimates can lead to elevated estimates of the rate parameter, here represented by *N*_e_ *E*(*s*). We observed this pattern in *A. lyrata*. This estimate is inherent to the type of distribution used to describe the DFE but does not directly have a biological explanation. However, we observe that very few sites actually have an extremely high *N*_e_ *s* value (>10,000 *N*_e_ *s*) due to the strongly leptokurtic gamma distribution (Figure S4). Instead of using the *N*_e_ *s* value per se, it is more meaningful to describe the shape of the distribution with frequency of *N*_e_ *s* values in bins (Keightley & Eyre‐Walker, 2007).

**Table 3:** Estimates of population size rescaling backward in time (N1/N2), mean selective effect (*N*_e_ *s*), and shape parameter (*β*) of fitness effect distribution

Species	N2/N1	*N*_e_ *E*(*s*) (95% C.I.)	*β* (95% C.I.)
*A. lyrata*	0.54	−2,281,880 (−12,192,657, −651,536)	0.07 (0.07, 0.08)
*C. grandiflora*	1.04	−454 (−473, −324)	0.23 (0.23, 0.24)

**Figure 3:**
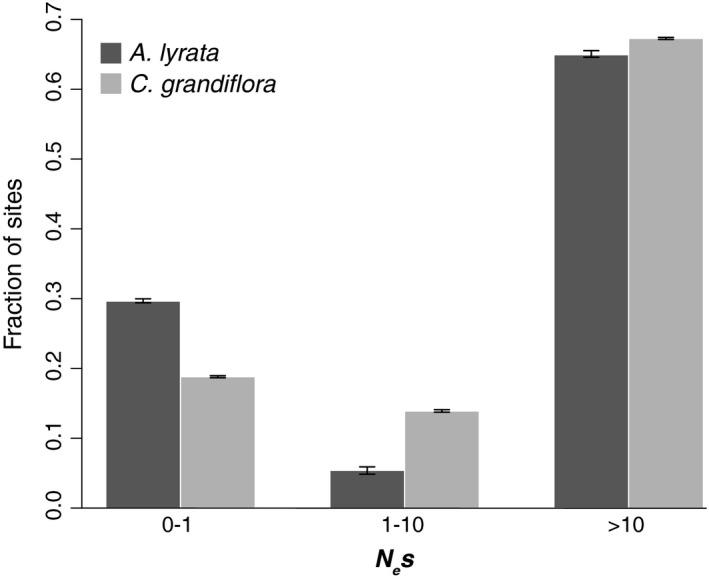
Estimated distribution of fitness effects for *A. lyrata* and *C. grandiflora* populations. The *X*‐axis shows the estimated population scaled selection coefficient (*N*_e_ *s*) binned in three categories: nearly neutral (*N*_e_ *s* < 1), intermediate (1 < *N*_e_ *s* < 10), and strongly deleterious (*N*_e_ *s* > 10). The *Y*‐axis shows the proportion of sites assigned into each category. Error bars show the 95% C.I. for the point estimates based on 100 bootstrap replicates

We found significant differences in the estimated DFE between species (Figure 3). In general, larger proportions of new mutations in 0‐fold sites were estimated to be effectively neutral (*N*_e_ *s* < 1) in *A. lyrata* (*A. lyrata*, 30%; *C. grandiflora*, 15%). Conversely, in *C. grandiflora* intermediate fitness effect (1 < *N*_e_ *s* < 10) or strongly deleterious (*N*_e_ *s* > 10) mutations were estimated to be more common; in *A. lyrata*, 5% and 65% of mutations were assigned to intermediate and strongly deleterious categories, respectively, while in *C. grandiflora*, the corresponding proportions were 14% and 71% (Figure 3).

### 3.4 Positive selection

The analysis of diversity and divergence at 0‐fold and 4‐fold degenerate sites indicated that 5.2% (3.7%–6.3%, 95% C.I.) of 0‐fold divergence was due to positive selection (*α*) in our *A. lyrata* population while in the *C. grandiflora* population the corresponding estimate was 18% (17%–20%, 95% C.I.). The estimates of proportion of adaptive substitutions relative to neutral divergence (*ω*) were 1.5% (1.1%–1.9%, 95% C.I.) for the *A. lyrata* population and 3.7% (3.5%–4.2%, 95% C.I.) for the *C. grandiflora* population.

### 3.5 Factors affecting genome‐wide diversity

Both study populations harbor high variation in diversity across the genome. Hence, we hypothesize that linked selection may contribute to the diversity patterns. To test this hypothesis, we studied the dependence of *π*_4_ on five explanatory variables: divergence at 4‐fold degenerate sites (*d*_4_), exonic bp, recombination rate, codon usage bias (*N*_c_'), and transposable element (TE) content in 50 kb windows. Under linked selection, we expect a positive correlation between recombination rate and diversity and negative correlation between diversity and the exon density, whereas no such correlation is expected in the absence of linked selection. Furthermore, Hill‐Robertson interference (Hill & Robertson, 1966) might also be expected to result in a reduced efficacy of weak selection in regions of low recombination and thus less codon usage bias in such regions (Kliman & Hey, 1993).

The best multiple linear regression model with model selection (BIC) explained 47% of the variation in diversity in the *C. grandiflora* dataset and 11% in the *A. lyrata* dataset (Table S2). In both species, recombination rate had significant positive effect on neutral diversity (Table 4), whereas exon density had significant negative effect on neutral diversity in *C. grandiflora*. In the *A. lyrata* dataset exon density also had negative effect on neutral diversity (Table S3) but it was not included in the best model (Table 4; Table S2). TE% had a positive effect whereas codon bias had a negative effect on neutral diversity in both populations studied. Comparing models including each explanatory variable separately indicated that for *C. grandiflora* recombination rate and for *A. lyrata* TE% explained the highest proportion of variance in neutral diversity and had the lowest BIC among the single variable models (Table S2). We observed significant correlation between the explanatory variables (Table S3) but variance inflation factor analysis did not indicate severe effect of multicollinearity (Table 3). Further, the distributions of residuals suggest that the model assumptions are met reasonably well (Figures S5 and S6), and only a small deviation from normality was observed (Table 4).

**Table 4:** Statistics for the best multiple regression models, based on a BIC model selection, explaining variance in *π*_4_ for populations of *A. lyrata* and *C. grandiflora*

Species	Variable	Coefficient	*SE*	*t*‐Value	*p*‐Value	√vif
*A. lyrata*	Intercept	−0.004	0.023	−0.184	0.854	–
	Rec. rate	0.146	0.023	6.427	<0.001	1.003
	*d*_4_	0.136	0.023	5.953	<0.001	1.005
	TE%	0.210	0.023	9.231	<0.001	1.004
	*N*_c_'	−0.102	0.023	−4.501	<0.001	1.004
*C. grandiflora*	Intercept	0	0.018	0	1	–
	Rec. rate	0.449	0.020	22.57	<0.001	1.107
	Exonic bp	−0.100	0.022	−4.56	<0.001	1.221
	*d*_4_	0.233	0.019	12.21	<0.001	1.062
	TE%	0.171	0.021	7.98	<0.001	1.190
	*N*_c_'	−0.095	0.020	−4.82	<0.001	1.092

### 3.6 Diversity around functional regions

**Figure 4:**
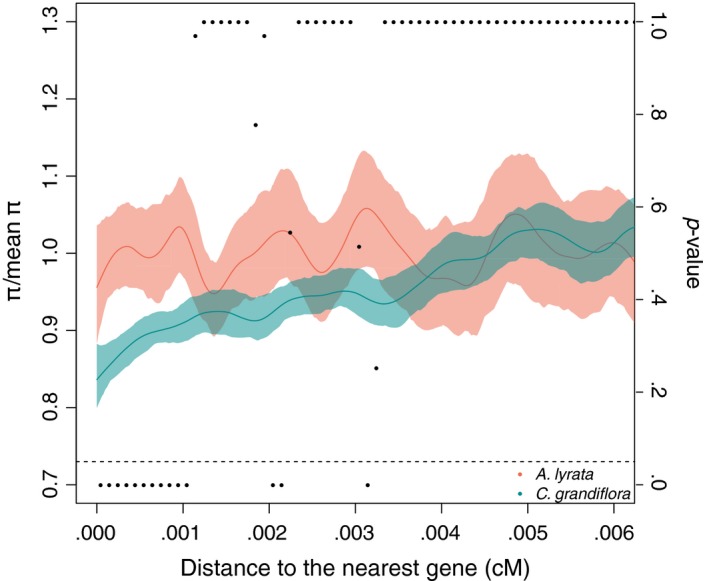
Diversity level as a function of distance from the nearest gene for *A. lyrata* (coral) and *C. grandiflora* (green) (95% C.I. based on 100 bootstrap replicates) and corresponding two‐sided *p*‐values (right *y*‐axis) for species difference in *π* based on comparison of 100 bootstrap pairs. *π* estimates are plotted as line, *p*‐values as points. The dashed line is showing the 0.05 *p*‐value cutoff

Selection on genes can also decrease diversity at nearby loci due to linkage disequilibrium. At equilibrium, a stronger decrease in diversity around genes is expected due to stronger efficacy of selection in populations of larger effective population size (Nordborg et al., 1996). In addition to this basic expectation, simulation results by Torres et al. (2019) suggest that after a strong population bottleneck, this diversity drop may even disappear over time. To test this prediction in our study populations, we studied diversity as a function of distance to the nearest gene. In the *A. lyrata* population, we found no evidence for reduced diversity near genes while in the *C. grandiflora* population there was a clear effect with intergenic regions near genes having 80% of the variation of the average intergenic variation (Figure 4). The difference was significant between the populations near genes (Figure 4). The effect of linked selection disappears approximately after 0.003–0.004 cM in *C. grandiflora* (Figure 4).

## 4 DISCUSSION

In this study, we examined patterns of genome‐wide diversity in coding and intergenic regions in populations of two outcrossing Brassicaceae species, *A. lyrata* and *C. grandiflora*. Our aim was to investigate the role of selection in shaping genome‐wide variation and more specifically to compare patterns of linked selection in two populations of species with similar mating system but differing in their demographic history.

The basic finding was that *C. grandiflora* had higher diversity in comparison with *A. lyrata*. In the *A. lyrata* dataset, we observed lower intergenic diversity in comparison with the diversity at the 4‐fold degenerate sites. This was not expected since under the assumption that intergenic regions evolve neutrally, the effect of direct and linked selection is expected to be weak. We also observed a higher proportion of low frequency variants at intergenic sites in both species which is not expected assuming that background selection affects the coding silent sites (4‐fold degenerate sites) more than sites distant from genes. A possible confounding factor that may cause these unexpected patterns is selection at intergenic sites. Indeed, many studies have suggested some degree of selection also in intergenic regions, especially close to genes (Andolfatto, 2005; Williamson et al., 2014; Woerner et al., 2018) although the strongest selection is usually found in coding regions. In addition, if mapping rate in the intergenic regions is low due to high divergence, this could cause frequent allelic dropout and hamper diversity estimation in these regions. This might be likely given the high repeat content of *A. lyrata* genome (Hu et al., 2011). However, after restricting our analyses to *A. lyrata* samples with only a slightly lower mapping rate, we still estimated a lower level of diversity in intergenic regions (Tables S4 and S5). Hence, it does not seem that this issue is responsible for the observed results.

In our demographic analysis, we attempted to limit the impact of linked selection by focusing on intergenic sites in regions of high recombination that were not close to genes. The most likely models suggested a strong effective population size decline starting more than 500,000 generations ago in the *A. lyrata* population and an effective population size increase starting about 1.4 million generations ago in the *C. grandiflora* population. These findings are generally in line with previous studies (*A. lyrata*: Mattila et al., 2017; Pyhäjärvi et al., 2012; Ramos‐Onsins, Stranger, Mitchell‐Olds, & Aguadé, 2004; Ross‐Ibarra et al., 2008: *C. grandiflora*: Douglas et al., 2015; Slotte et al., 2010, St. Onge et al., 2011). Hence, this dataset offered a good contrast for studying the effect of demography on genome‐wide selection in these species.

We contrasted the strength of purifying and positive selection in these populations by comparing patterns of diversity and divergence at 0‐fold and 4‐fold degenerate sites. The higher *π*_0_/*π*_4_ in *A. lyrata* suggested relaxed efficacy of selection but can also be due to differences in dynamics of diversity recovery after population size change (Comeron, 2017; Torres et al., 2019). For instance, although at equilibrium we expect a decreased ratio of diversity at selected versus neutral sites, after a population size increase sites under stronger background selection reach equilibrium diversity faster than neutrally evolving sites (Comeron, 2017) and thus this selected versus neutral ratio may even increase at first.

To explicitly test selection strength, we estimated distribution of fitness effects of new mutations at 0‐fold degenerate sites using a method that takes into account the demographic history. We found that the *C. grandiflora* population had more efficient purifying selection than the *A. lyrata* population. This is in line with the general prediction on the impact of effective population size on the efficacy of selection (Eyre‐Walker & Keightley, 2007; Ohta, 1973). The general observation of reduced purifying selection associated with smaller effective population size has been found also in other plant species, for example in *Populus*, *Helianthus* and *Lactuca* (Strasburg et al., 2011; Wang, Street, Scofield, & Ingvarsson, 2016). Other factors such as life‐history traits have also been shown to correlate with the DFE (Chen, Glémin, & Lascoux, 2017). In plants, the strongest correlates were mating system and longevity, with selfing and long‐lived species having weaker purifying selection in comparison with outcrossing annual species. In addition, longevity has also been found to be associated with lower diversity (Chen et al., 2017; Romiguier et al., 2014) which may be due to different life‐history strategies among short‐ and long‐lived species such as between annual *C. grandiflora* and perennial *A. lyrata*. Nevertheless, we found large differences in the DFE as well as in diversity between two outcrossing species both having relatively short lifespan but strong differences in the demographic histories. We hence suggest that demographic history is likely the ultimate cause of these observed patterns although it might be linked to different life‐history traits. Laenen et al. (2018) also studied the combination of demographic and mating system effects on the DFE and found reduced diversity and relaxed purifying selection in selfing, strongly bottlenecked populations of *Arabis alpina*. In contrast, mixed mating populations had a similar level of diversity and purifying selection as outcrossing populations. They concluded that effective population size reduction associated with the transition to selfing is important to explain the observed difference in purifying selection.

Our estimates of the proportion of positively selected fixations at 0‐fold degenerate sites also suggest a smaller contribution of positive selection in the *A. lyrata* population compared to the *C. grandiflora* population. These results are consistent with previous studies in *C. grandiflora* (Slotte et al., 2010, 2013; Williamson et al., 2014) and *A. lyrata* (Foxe et al., 2008; Gossmann et al., 2010) and support generally stronger efficacy of selection in *C. grandiflora*.

We note that although the species are similar in many respects except the contrasting demographic histories, they also have differences with respect to their genome content, with, for example, shorter intergenic distances and fewer TEs in the *Capsella* genome (Hu et al., 2011; Slotte et al., 2013). Additionally, the effect of positive selection has been found to be stronger in *C. grandiflora* (Gossmann et al., 2010) which may also affect the diversity patterns. In turn, the recent colonization history of the studied *A. lyrata* population has been associated with local adaptation (Leinonen et al., 2009) which is likely to have shaped patterns of diversity at adaptive loci (Hämälä & Savolainen, 2019; Mattila et al., 2016; Mattila et al., 2017; Toivainen, Pyhäjärvi, Niittyvuopio, & Savolainen, 2014). However, the estimated proportion of adaptive substitutions relative to neutral divergence was <5% in both species. Hence, we suggest that background selection has likely stronger impact on the diversity in these study populations.

### 4.1 Linked selection shapes the patterns of genome‐wide variation in *A. lyrata* and *C. grandiflora*

Understanding the magnitude and factors contributing to the linked selection is important for evolutionary inferences using population genetics data (Burri, 2017; Comeron, 2017). We first investigated the role of linked selection for patterns of genome‐wide diversity using linear modeling, while accounting for the impact of a set of genomic features that might also affect diversity levels. Previous theoretical modeling of background selection in *A. lyrata* has suggested that the impact of background selection is expected to vary across the *A. lyrata* genome (Slotte, 2014). The basic theoretical prediction on linked selection is a positive correlation between diversity and recombination rate (Begun & Aquadro, 1992; Kaplan et al., 1989; Thomson, 1977). In addition, variation in the frequency of selected sites along the genome, for example due to variation in gene density, can give rise to a negative relationship between diversity and gene density (Slotte, 2014). In this study, regression analysis confirmed independent effects of recombination rate and exonic bp on neutral diversity in both populations studied, suggesting that diversity is shaped by linked selection. The variables explaining the highest proportion of variance in neutral diversity were for *C. grandiflora* recombination rate and TE% for *A. lyrata*, both of which were associated with increased diversity. The large effect of TE% may be due to generally lower functional constraint in these regions, which allows accumulation of TEs as well as higher variation. In both study populations, effective number of codons was negatively associated with diversity, which may be due to differences in the strength of selection for optimal codon usage. In *C. grandiflora*, the number of exonic bp per window had a negative effect on diversity, as expected under background selection (Slotte, 2014), but it was not included in the best model for *A. lyrata*. This is likely due to overall lower diversity in *A. lyrata* and hence decreased explanatory power in the regression analysis.

In contrast to our results, a previous study testing for linked selection in *A. lyrata* found no evidence for a correlation between neutral diversity and recombination rate (Wright et al., 2006). However, the correlation in *A. lyrata* was relatively weak in comparison with that in *C. grandiflora*. Hence, the difference between previous work and the current study may be due to the small number of loci studied previously. Therefore, our large dataset allowed us to detect small effects undetectable in previous studies.

### 4.2 Diversity around genes

To inspect the effect of linked selection further, we studied the level of diversity as a function of distance from annotated protein coding genes. Under linked selection diversity is expected to decrease near genes but the pattern and magnitude of this effect are expected to be affected by demographic history and especially the time since the shift in the effective size of the population (Comeron, 2017; Torres et al., 2019). We found a decrease in diversity in the flanking regions of genes in *C. grandiflora* but not in *A. lyrata*. We further observed higher uncertainty in the diversity estimates for *A. lyrata* which likely reflects higher sampling variance in the population with overall lower genetic diversity. The likely causal factor explaining the differences in linked selection between the species is the difference in the demographic history. The observed pattern in the *A. lyrata* population was somewhat surprising given that regression analysis suggested an effect of linked selection.

To compare our results with the expected patterns of diversity in nonequilibrium populations, we investigated the simulation results of Torres et al. (2019) in detail. For comparison, we scaled the timing of the demographic events by the ancestral effective population size. Using the estimated model for *A. lyrata*, the scaled time since the first size decrease was 0.8 *N*_ANC_ while the second was 0.002 *N*_ANC_ (maximum bootstrap value 0.04 *N*_ANC_) ago. This indicates that although the first population size decrease happened relatively long ago, our *A. lyrata* population has not had much time to recover from the second decrease which was also estimated to be very drastic (*N*_e_ decrease from almost 200,000 to only 6,000). Immediately after a strong population size decrease, the expected reduction in diversity due to linked selection around deleterious loci is expected to be weak and it disappears over time (Torres et al., 2019). Since the diversity in our *A. lyrata* population is likely impacted by an ancient and a more recent bottleneck, a flat relationship of distance from genes and diversity is at least qualitatively in line with the simulation results and suggests that drift is mainly dominating diversity patterns at intergenic sites in *A. lyrata*. In addition, the population has had short time to recover from the more recent population size change and the effect of selection on diversity is still expected to decrease if the population size remains the same.

For *C. grandiflora*, the scaled time since the most recent population size change (a modest population size increase) was 0.6 *N*_ANC_, suggesting that this population is closer to equilibrium. However, for *C. grandiflora* the uncertainty is much larger and the estimated scaled time since the last increase ranged from 0.01 to 1.7 *N*_ANC_. In such a demographic history, diversity is expected to dip around genes (Torres et al., 2019). In *C. grandiflora*, the observed magnitude of diversity reduction is comparable with results from simulations (Torres et al., 2019) and empirical results in teosinte and maize (Beissinger et al., 2016). It should however be noted that after population size expansion, diversity in regions under background selection may actually increase relative to that under neutrality, despite the theoretical expectation that population size increase should lead to a stronger impact of linked selection (Torres et al., 2019). Indeed, in their simulations, Torres et al. (2019) observed that it could take up to 10 *N*_ANC_ generations to reach equilibrium diversity level after a population size expansion.

## 5 CONCLUSIONS

Despite the similar mating system of *A. lyrata* and *C. grandiflora* populations studied here, they differ drastically in their patterns of variation, demographic history, and selection; we estimated that the *C. grandiflora* population had stronger purifying selection, higher proportion of sites under positive selection, and more pronounced decrease of diversity around genes due to linked selection. In the *A. lyrata* population, we do not observe a reduction in diversity near genes in comparison with the regions distant from genes. This observation is consistent with simulation‐based results and we hence conclude that nonequilibrium demographic history is likely to have modified the typical signature of linked selection close to genes in the studied *A. lyrata* population. However, the positive correlation of recombination rate and neutral diversity suggests that linked selection has had an effect on diversity to some degree in both study populations. Hence, examining further the effects of linked selection will be of utmost importance to understand differences in genetic diversity in natural populations.

## CONFLICT OF INTEREST

None declared.

## AUTHOR CONTRIBUTIONS

TMM, BL, OS, and TS designed the study. TH provided data. TMM, BL, and RH conducted the data analysis. TMM and TS wrote the manuscript with input from all the other authors.

## Data Availability

The analyzed sequence data are available at the European Bioinformatics Institute for the *C. grandiflora* dataset (project accession number PRJEB12072) and for *A. lyrata* at NCBI Sequence Read Archive (BioSample accession numbers SAMN06141173–SAMN06141177 and SAMN09069416–SAMN09069422). Input files for DFEalpha and regression analyses are available from Figshare (*A. lyrata* DFEalpha file: https://figshare.com/s/c929239b54f562d2cdfe; *C. grandiflora* DFEalpha file: https://figshare.com/s/163d79dde9067de6e2e4; regression analyses script: https://figshare.com/s/50e95b4f0d49b0efe662; regression analyses *A. lyrata* data: https://figshare.com/s/41e6a2762207b60c4557; regression analyses *C. grandiflora* data: https://figshare.com/s/06641a1a9e366d2acf83).
