# Supplementary material for: Impact of demography on linked selection in two outcrossing Brassicaceae species
Source: Ecol Evol. 2019 Aug 13;9(17):9532–45. doi: 10.1002/ece3.5463 (PMC6745670; doi:10.1002/ece3.5463)
Supplement: Supplementary file 1 [file ECE3-9-9532-s001.docx]

SUPPORTING INFORMATION

Article title: Impact of demography on linked selection in two outcrossing Brassicaceae species

Authors: Tiina M. Mattila, Benjamin Laenen, Robert Horvath, Tuomas Hämälä, Outi Savolainen, Tanja Slotte

TABLE S1 Estimated demographic parameters (95 % C.I. in parenthesis) for the study populations of *A. lyrata (A. l.)* and *C. grandiflora (C. g.)*. Parameter estimates for the best-fit models are highlighted in bold.

| Species | Model | lnL | AIC | *N_ANC_* | *N_CUR_* | Time | *N_BOT_* | T_BOT_ | BOT duration |
| --- | --- | --- | --- | --- | --- | --- | --- | --- | --- |
| *A. l.* | Constant | -4167.98 | 8341.9 | NA | 237001  (235227–  238888) | NA | NA | NA | NA |
|  | two-epoch | -266.99 | 539.98 | 340235  (334580–  347083) | 26570  (16055–  36040) | 10415  (5734–  15283) | NA | NA | NA |
|  | growth | -249.25 | 504.5 | 374764  (338395– 385685) | 3828  (3254–  30564) | 3206  (2749–  41164) | NA | NA | NA |
|  | bottlegrowth | -249.13 | 506.25 | 375282  (361660–  389773) | 3069  (2899–  4323) | 2569  (2333–  3753) | 300312  (270968–  452143) | NA | NA |
|  | **three-epoch** | **-242.56** | **495.11** | **626113**  **(340036–**  **982622)** | **6355**  **(3469–**  **27260)** | **NA** | **195370**  **(125552– 274912)** | **1089**  **(514– 10213)** | **520418**  **(6565– 818359)** |

TABLE S1 Continued

| Species | Model | lnL | AIC | N_ANC_ | N_CUR_ | Time | N_BOT_ | T_BOT_ | BOT duration |
| --- | --- | --- | --- | --- | --- | --- | --- | --- | --- |
| *C. g.* | constant | -8025 | 16056.01 | NA | 1010641  (1006096– 1014335) | NA | NA | NA | NA |
|  | two-epoch | -100.94 | 207.88 | 613507 (596233–  631537) | 1528928  (1507810–  1547917) | 980338 (916478–  1056222) | NA | NA | NA |
|  | growth | -108.68 | 223.36 | 309253 (306723–  332639) | 1677981  (1664528– 1692661) | 3702373 (3521997–  3738424) | NA | NA | NA |
|  | **bottlegrowth** | **-98.24** | **204.48** | **374849 (265803–**  **1916547)** | **1615595**  **(1525195– 1667344)** | **2117864 (1017408–**  **7611384)** | **789565 (56206–**  **1499766)** | **NA** | **NA** |
|  | **three-epoch** | **-97.84** | **205.68** | **518572 (281865–**  **26285211)** | **1578151**  **(1431263– 3051412)** | **NA** | **1312595 (350255–**  **1501449)** | **302395 (6219–**  **1375337)** | **1114200**  **(789–**  **11617340)** |

lnL = log-likelihood, AIC = Akaike Information Criterion, N = effective population size, T = generations ago, ANC = ancestral, CUR = current, BOT = bottleneck

##### Table S2 BIC values and explanatory power of the different multiple regression models explaining the variance in π_4_ for the *A. lyrata* and *C. grandiflora* population. BIC values and explanatory power for the best models are highlighted in bold.

| Model | BIC | Explanatory power |
| --- | --- | --- |
| *A. lyrata* |  |  |
| **rec. rate + *d_4_* + TE % + N_c_'** | **4597.614** | **10.74%** |
| Full model | 4604.003 | 10.80% |
| rec. rate + *d_4_* + TE % | 4610.383 | 9.67% |
| rec. rate + *d_4_* + TE % + exon bp | 4616.345 | 9.75% |
| rec. rate + TE % + N_c_' | 4625.356 | 8.87% |
| *d_4_* + TE % + N_c_' | 4631.111 | 8.56% |
| rec. rate + TE % + N_c_' + exon bp | 4631.764 | 8.92% |
| rec. rate + TE % | 4634.797 | 7.95% |
| *d_4_* + TE % + N_c_' + exon bp | 4635.651 | 8.71% |
| rec. rate + *d_4_* + N_c_' + exon bp | 4639.81 | 8.49% |
| rec. rate + TE % + exon bp | 4640.82 | 8.03% |
| *d_4_* + TE % | 4644.402 | 7.43% |
| *d_4_* + TE % + exon bp | 4648.238 | 7.63% |
| rec. rate + *d_4_* + exon bp | 4653.179 | 7.36% |
| TE % + N_c_' | 4662.822 | 6.42% |
| TE % + N_c_' + exon bp | 4667.282 | 6.58% |
| *d_4_* + N_c_' + exon bp | 4667.862 | 6.55% |
| rec. rate + N_c_' + exon bp | 4669.637 | 6.45% |
| TE % | 4672.583 | 5.46% |
| rec. rate + *d_4_* + N_c_' | 4673.553 | 6.23% |
| TE % + exon bp | 4676.399 | 5.66% |
| rec. rate + exon bp | 4679.563 | 5.49% |
| *d_4_* + exon bp | 4681.432 | 5.38% |
| rec. rate + *d_4_* | 4691.177 | 4.83% |
| N_c_' + exon bp | 4701.404 | 4.26% |
| rec. rate + N_c_' | 4704.832 | 4.06% |
| *d_4_* + N_c_' | 4710.13 | 3.76% |
| exon bp | 4711.36 | 3.27% |
| rec. rate | 4718.663 | 2.85% |
| *d_4_* | 4728.47 | 2.29% |
| N_c_' | 4745.834 | 1.28% |
| *C. grandiflora* |  |  |
| **Full model** | **3653.107** | **47.20%** |
| rec. rate + *d_4_* + TE % + N_c_' | 3666.438 | 46.53% |
| rec. rate + *d_4_* + TE % + exon bp | 3668.937 | 46.45% |
| rec. rate + *d_4_* + TE % | 3682.531 | 45.76% |
| rec. rate + *d_4_* + N_c_' + exon bp | 3708.416 | 45.14% |
| rec. rate + *d_4_* + exon bp | 3735.976 | 43.96% |
| rec. rate + *d_4_* + N_c_' | 3783.393 | 42.31% |
| rec. rate + TE % + exon bp | 3788.135 | 42.14% |
| rec. rate + TE % + N_c_' + exon bp | 3788.94 | 42.38% |
| rec. rate + *d_4_* | 3819.571 | 40.76% |
| rec. rate + TE % | 3831.561 | 40.32% |
| rec. rate + TE % + N_c_' | 3833.604 | 40.51% |
| rec. rate + N_c_' + exon bp | 3841.72 | 40.22% |
| rec. rate + exon bp | 3847.762 | 39.73% |
| rec. rate + N_c_' | 3980.903 | 34.62% |
| rec. rate | 3989.903 | 33.96% |
| *d_4_* + TE % + N_c_' + exon bp | 4090.534 | 30.73% |
| *d_4_* + TE % + N_c_' | 4130.514 | 28.69% |
| *d_4_* + N_c_' + exon bp | 4152.149 | 27.75% |
| *d_4_* + TE % + exon bp | 4200.236 | 25.59% |
| *d_4_* + TE % | 4246.249 | 23.13% |
| TE % + N_c_' + exon bp | 4247.068 | 23.43% |
| *d_4_* + N_c_' | 4288.921 | 21.10% |
| *d_4_* + exon bp | 4292.527 | 20.92% |
| N_c_' + exon bp | 4306.885 | 20.23% |
| TE % + exon bp | 4313.664 | 19.90% |
| TE % + N_c_' | 4338.24 | 18.69% |
| exon bp | 4398.462 | 15.26% |
| TE % | 4404.753 | 14.93% |
| *d_4_* | 4469.693 | 11.49% |
| N_c_' | 4542.504 | 7.47% |

##### Table S3 Observed correlations between π_4_ and the five explanatory variables: divergence at 4-fold degenerate sites (*d_4_*), exonic bp, recombination rate, codon usage bias (N_c_') and transposable element (TE) content in 50 Kb windows. *P*-value were corrected for multiple testing using a Benjamini and Hochberg *p*-value adjustment.

|  | *A. lyrata* | | | *C. grandiflora* | |
| --- | --- | --- | --- | --- | --- |
| Explanatory variable | | Pearson correlation coefficient | *p*-value | Pearson correlation coefficient | *p*-value |
| π_4_ & rec. rate | | 0.1622976 | 5.54*10^-11^ | 0.5827941 | 1.91*10^-148^ |
| π_4_ & *d_4_* | | 0.1666204 | 1.96*10^-11^ | 0.3389906 | 7.48*10^-45^ |
| π_4_ & exon bp | | -0.1764779 | 1.30*10^-12^ | -0.3906138 | 3.85*10^-60^ |
| π_4_ & TE % | | 0.2293338 | 9.10*10^-21^ | 0.3864177 | 6.76*10^-59^ |
| π_4_ & N_c_' | | -0.1098957 | 1.25*10^-5^ | -0.2732845 | 3.18*10^-29^ |
| rec. rate & *d_4_* | | 0.0601738 | 1.99*10^-2^ | 0.1388762 | 1.92*10^-8^ |
| rec. rate & exonic bp | | -0.1158486 | 4.40*10^-6^ | -0.2741532 | 2.34*10^-29^ |
| rec. rate & TE % | | 0.04753527 | 6.31*10^-2^ | 0.2435108 | 2.31*10^-23^ |
| rec. rate & N_c_' | | -0.01720695 | 0.48 | -0.3379336 | 1.21*10^-44^ |
| *d_4_* & exonic bp | | -0.03171083 | 0.21 | -0.2835003 | 2.54*10^-31^ |
| *d_4_* & TE % | | 0.04653943 | 6.41*10^-2^ | 0.1442193 | 5.68*10^-9^ |
| *d_4_* & N_c_' | | 0.0555814 | 3.03*10^-2^ | 0.103215 | 2.85*10^-5^ |
| exonic bp & TE % | | -0.6233168 | 1.13*10^-181^ | -0.5174666 | 5.45*10^-112^ |
| exonic bp & N_c_' | | 0.0767033 | 2.97*10^-3^ | 0.134109 | 5.45*10^-8^ |
| TE % & N_c_' | | -0.06617953 | 1.07*10^-2^ | -0.217914 | 6.35*10^-19^ |


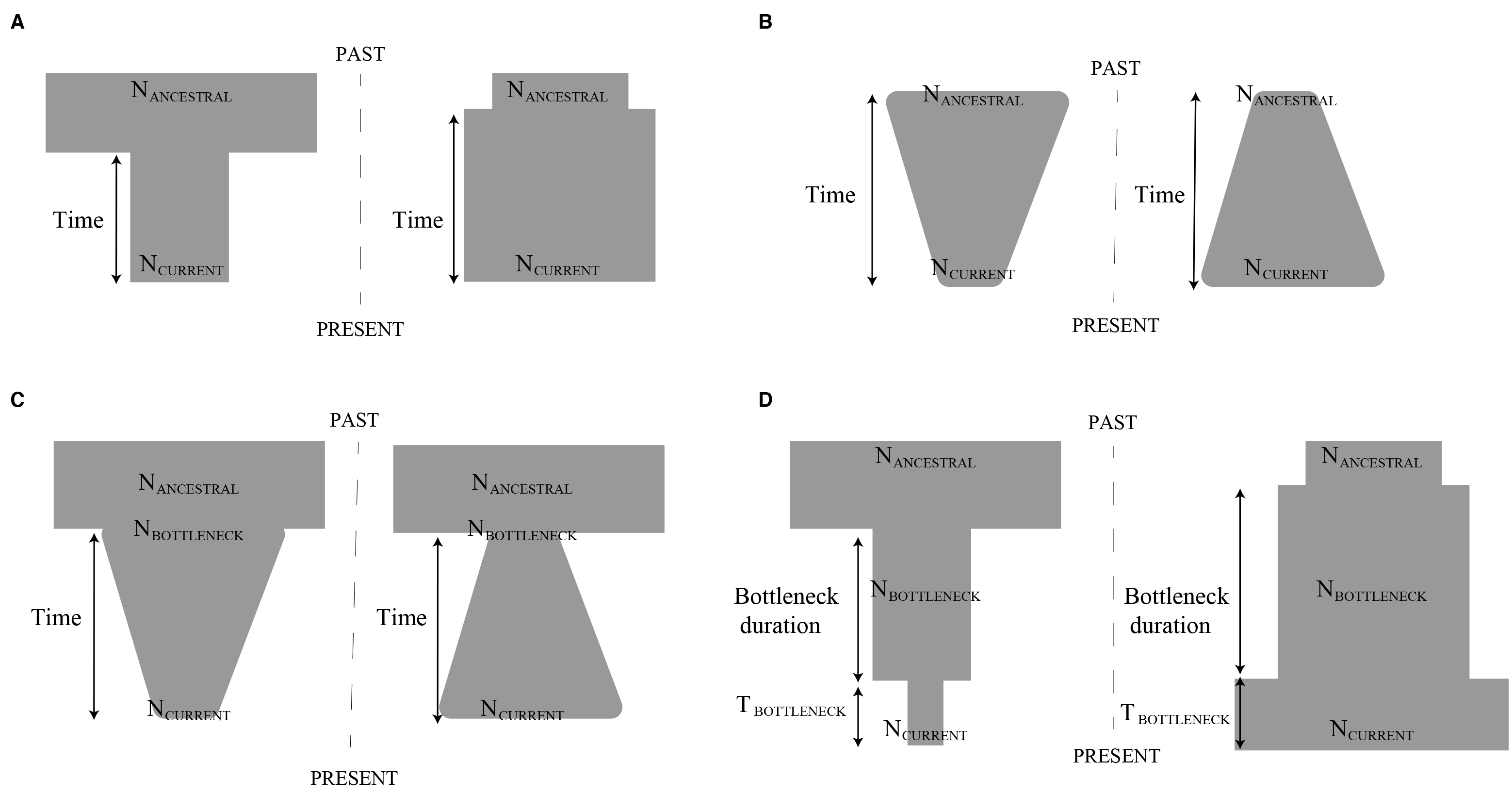


Figure S1 Schematic representation of four non-equilibrium demographic models used in this study **A** two-epoch model **B** growth model **C** bottlegrowth model and **D** three-epoch model. For each model, the graph shows examples of either increase or decrease in the effective population size.

Figure S2 Recombination rate estimates along the A. lyrata genome

Figure S3 Recombination rate estimates along the Capsella genome. Recombination rate estimates are from a genetic map between C. rubella and C. grandiflora.


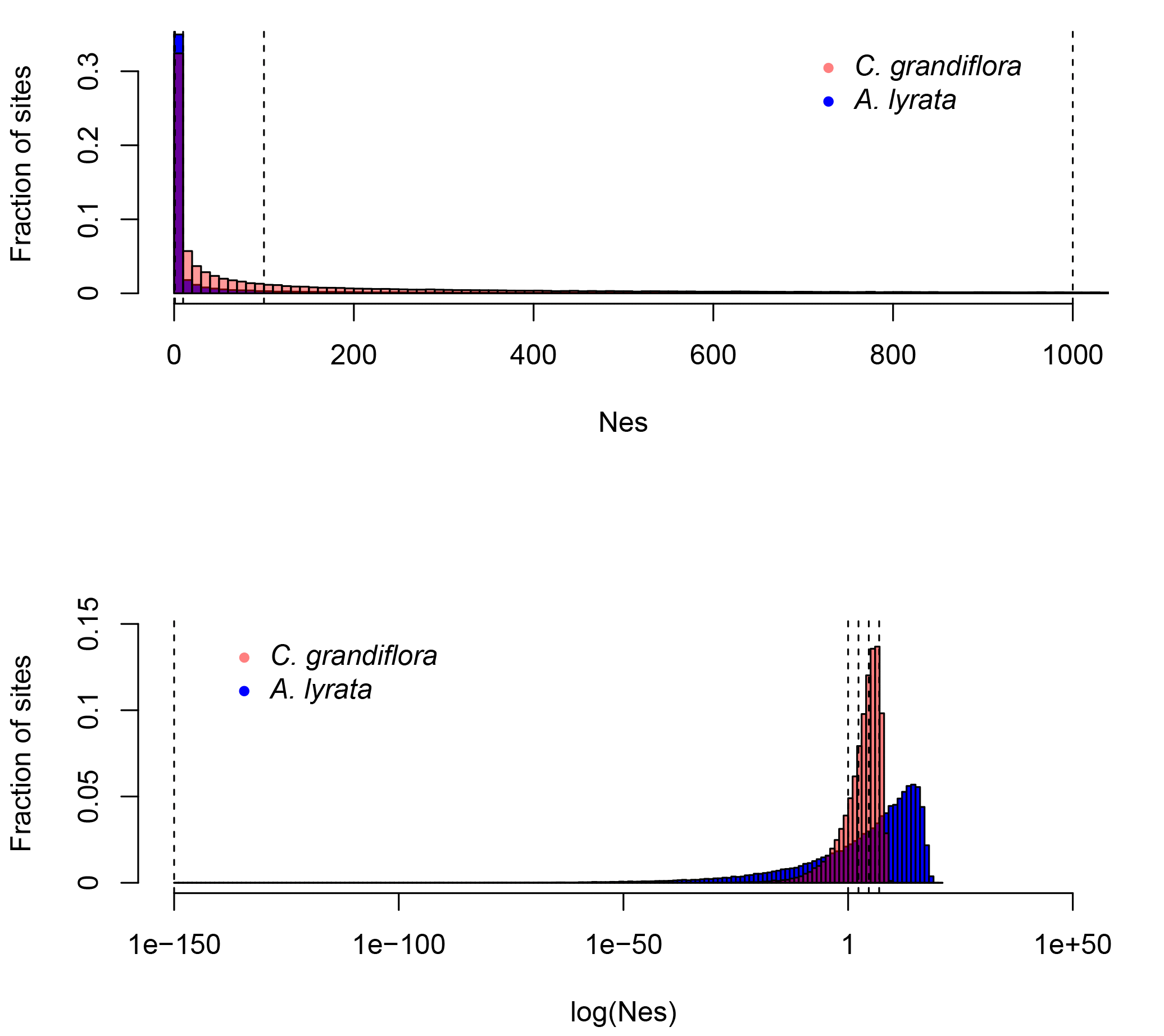


Figure S4. Distribution of population scaled fitness effects of new mutations (Nes) for *A. lyrata* and *C. grandiflora* (Upper panel) (bin size 10). The lower panel shows the distribution on logarithmic scale. The dashed vertical lines show Nes intervals 0-1, 1-10, 10-100 and 100-1000.

Figure S5 Diagnostic plots of the best multiple regression models based on a BIC model selection explaining variance in *π_4_* for the *A. lyrata* population.

Figure S6 Diagnostic plots of the best multiple regression models based on a BIC model selection explaining variance in *π_4_* for the *C. grandiflora* population.
